# PhIP-Seq: methods, applications and challenges

**DOI:** 10.3389/fbinf.2024.1424202

**Published:** 2024-09-04

**Authors:** Ziru Huang, Samarappuli Mudiyanselage Savini Gunarathne, Wenwen Liu, Yuwei Zhou, Yuqing Jiang, Shiqi Li, Jian Huang

**Affiliations:** ^1^ School of Life Science and Technology, University of Electronic Science and Technology of China, Chengdu, China; ^2^ School of Healthcare Technology, Chengdu Neusoft University, Chengdu, China

**Keywords:** phage, PhIP-Seq, antibody, immunity, biotechnological applications

## Abstract

Phage-immunoprecipitation sequencing (PhIP-Seq) technology is an innovative, high-throughput antibody detection method. It enables comprehensive analysis of individual antibody profiles. This technology shows great potential, particularly in exploring disease mechanisms and immune responses. Currently, PhIP-Seq has been successfully applied in various fields, such as the exploration of biomarkers for autoimmune diseases, vaccine development, and allergen detection. A variety of bioinformatics tools have facilitated the development of this process. However, PhIP-Seq technology still faces many challenges and has room for improvement. Here, we review the methods, applications, and challenges of PhIP-Seq and discuss its future directions in immunological research and clinical applications. With continuous progress and optimization, PhIP-Seq is expected to play an even more important role in future biomedical research, providing new ideas and methods for disease prevention, diagnosis, and treatment.

## 1 Introduction

Antibodies, also known as immunoglobulins (Igs), are proteins produced by the adaptive immune system that play a crucial role in providing defense against pathogens and foreign substances. Antibodies can recognize and bind to pathogens and foreign substances called antigens in the body, neutralizing them and marking them for destruction by other immune cells (Sun et al., 2020). The unique antibody profile of each individual holds significant biological information including details about environmental exposures, allergic reactions, autoimmune disease processes, and responses to immunomodulatory therapies. This detailed information can be critical for understanding an individual’s immune status, diagnosing a variety of diseases, and guiding therapeutic interventions.

Traditional antibody binding assays, such as ELISA, have been a cornerstone in the field of immunology, providing critical insights into the recognition and response to various antigens. Western Blot (WB), also known as immunoblotting (Renart et al., 1979) is used to detect and semiquantify target proteins, making it a commonly used technique for antibody validation (Sule et al., 2023). However, traditional methods can only assess antibodies against a limited number of antigens. This limitation also hinders the determination of an individual’s full antibody profile and the identification of novel or uncharacterized antigens (Mohan et al., 2018). Moreover, the protein expression and purification involved in these tests can be laborious and resource-intensive, particularly for complex or poorly characterized antigens.

Bacteriophages are the most abundant and diverse class of viruses in the world, renowned for their simple construction and easy manipulation, making them ideal model organisms for biological research. In 1985, Professor George Smith invented phage display for studying antibody-peptide interactions (Smith, 1985). The coding sequences of target proteins or peptides are inserted into the phage vectors, allowing them to be displayed or presented on the surface of phage particles. The importance of phage display platforms was recognized with the awarding of the Nobel Prize to George P. Smith and Sir Gregory P. Winter for the ‘phage display of peptides and antibodies’ in 2018. Phage display libraries are widely used to identify novel antigen-specific monoclonal antibodies and map the binding sites or epitopes of proteins or antibodies. Phage display technology has also emerged as a robust platform for drug discovery in biotechnology due to its ability to facilitate the efficient identification of antibodies *in vitro* (Nagano and Tsutsumi, 2021). This technology facilitates extensive applications in biomedical research, drug development, and bioengineering (Kim et al., 2011).

One key advancement that has greatly improved the efficiency and scalability of phage display technology is Oligonucleotide Library Synthesis (OLS). OLS allows for the high-throughput synthesis of numerous oligonucleotide sequences, which can then be used to create highly diverse peptide libraries. Phage Immunoprecipitation Sequencing (PhIP-Seq) is a technique derived from traditional phage display that utilizes OLS to encode peptide libraries. PhIP-Seq enables the efficient detection of antibodies on a large scale by employing a high-capacity phage display peptide library, antibody immunoprecipitation, and high-throughput sequencing (Mohan et al., 2018). This technique can detect antibodies against hundreds of thousands of peptides and provide a comprehensive mapping of antibody profiles, making it particularly advantageous in studying complex diseases or immune responses to large pathogen families. Moreover, its high multiplexing capability allows for the analysis of multiple samples simultaneously, significantly reducing the cost of DNA sequencing per sample and enabling the analysis of large sets of samples. With these aforementioned characteristics, PhIP-Seq facilitates the discovery of novel antibodies and enhances our understanding of the immune response.

**FIGURE 1:**
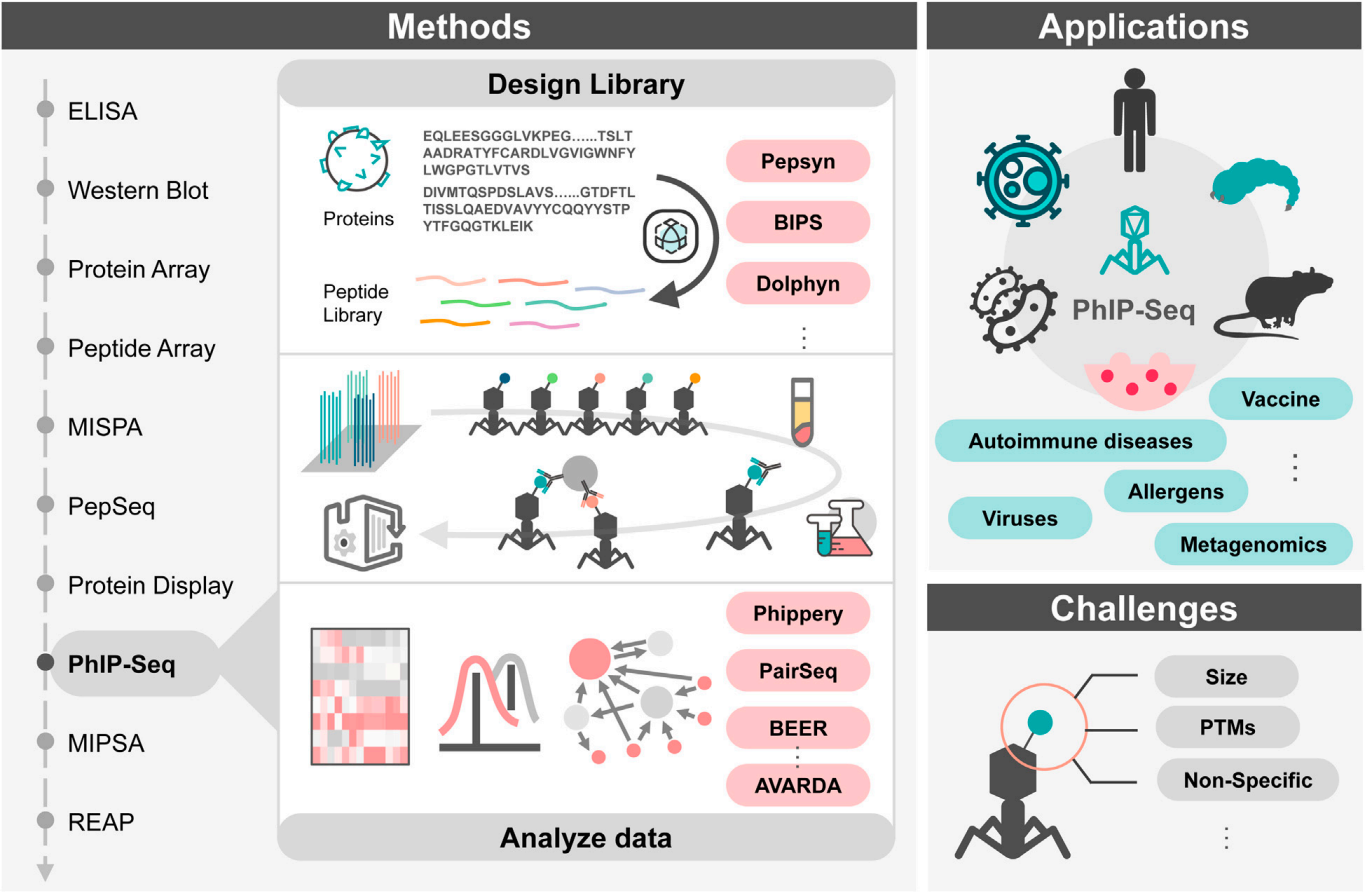
Graphical abstract. Left: Different technologies of antibody detection. Middle: Process overview and related tools for PhIP-Seq technology. Right: Applications and challenges of PhIP-Seq technology. Abbreviations: ELISA, Enzyme-linked Immunosorbent Assay; MISPA, Multiplexed In-Solution Protein Array; MIPSA, Molecular Indexing of Proteins by Self-Assembly; REAP, Rapid Extracellular Antigen Profiling; PTMs, Post-translational modifications.

As shown in Figure 1, In this review, we highlight the research progress of PhIP-Seq technology, encompassing the entire process from the library’s design to the data analysis. We have also summarized current challenges and potential solutions for them. Furthermore, we explore prospective directions for future development in PhIP-Seq technology and its applications.

## 2 Adaptation for human autoantibody profiling

Antibodies can recognize and bind to a wide range of biomolecular targets with specificity. Although they are responsible for providing immunity against pathogens, some antibodies referred to as autoantibodies, bind to self-antigens. These autoantibodies can cause different biological effects, such as modifying the body’s immune response and inducing damaging inflammation in various tissues (Jaycox et al., 2024). In many autoimmune diseases, numerous autoantibodies remain unidentified. Experimental techniques are constantly being developed to uncover novel autoantigens. Protein arrays, peptide arrays, and technologies such as MIPSA, PhIP-Seq, and REAP are some of the high-throughput methods that have significantly advanced the field of autoantibody profiling and autoimmune disease research (Carlton et al., 2023) (Table 1).

**TABLE 1:** The principles, advantages, and limitations for antibody profiling technologies.

	Principle	Antigen	Detection	Multi-flexability	Advantages	Limitations
ELISA	Detect and quantify specific antibodies or antigens by using antigen-antibody interactions and enzyme signal amplification	Whole proteins or subdomains of interest	Enzyme-tagged	Limited	Easily applicable standard laboratory test	Single antigen assay; time-consuming
Western Blot	Electrophoretic separation and immunodetection of proteins	Denatured proteins	Enzyme-tagged	Limited	Able to determine molecular weight and presence or absence of proteins, more sensitive	Relatively low throughput and longer and more complicated to operate
Protein Array	Probe molecules labeled with fluorescent dyes are added to the array, and protein interactions generate fluorescent signals	A complete protein fragment or domain	Fluorescent-tagged	Medium	Parallel detection of multiple proteins can be utilized for protein interaction and determining expression levels	Lack of post-translation modification and increased costs
Peptide Array	Similar to Protein Array	Peptide	Fluorescent-tagged	Medium	It can be used for rapidly screening protein binding sites, identifying peptide affinity, etc	Cross-reactivity may exist, and specificity needs to be considered
MISPA	Employs a solution-based protein library, with each protein antigen covalently linked to a unique DNA barcode	Full-length proteins	NGS	High	The ability to detect antibodies to conformational epitopes	Expanding the range of antigens has a higher cost
PepSeq	*in vitro* synthesis of a customizable peptide/DNA conjugate library	Peptide	NGS	High	Allowing for rapid, sensitive, and cost-effective analysis of antibody reactivity across a wide range of peptides using minimal sample input	Lack of post-translational modifications
Protein Display	Function by transforming cells with DNA libraries, allowing the encoded peptides to be displayed on the surface of cells or bacteriophage particles	Peptide	Sanger or NGS	High	Can display protein libraries enable the easy screening and identification of proteins that interact with target proteins	Limited by the construction and display efficiency of the protein library
PhIP-Seq	Encode the peptide library and display it on phages, followed by immunoprecipitation and subsequent NGS	Peptide	NGS	High	The ability to encode a library enables the selection of the antigenic protein group of interest	Lack of post-translational modifications and conformational epitopes of proteins
MIPSA	Displays full-length proteins on ribosomes, each with a unique DNA barcode for parallel sequencing	Full-length proteins	NGS	High	Can detect autoantibodies against conformational and discontinuous epitopes	Lack of post-translational modifications
REAP	Utilizing yeast cells as eukaryotic expression hosts, it displays a panel of extracellular proteins, each genetically barcoded for precise identification *via* NGS.	Extracellular and secreted proteins	NGS	High	Eukaryotic expression host results in antigen folding and post-translational modifications more like humans	The absence of human-like glycosylation patterns and the incomplete representation of the human proteome

Protein arrays enable parallel analysis of protein-protein interactions. They can be produced through cellular expression, using hosts such as bacteria, or yeast, or through cell-free methods such as *in situ* synthesis. The HuProt™ microarray v2.0, for instance, contains 19,394 proteins from 15,275 genes and has been used for antibody profiling in various diseases (Hu et al., 2012; Yang et al., 2016; Hu et al., 2017). Cell-free methods such as NAPPA and M-NAPPA offer a quick and adaptable alternative by synthesizing proteins directly from DNA on the array (Yu et al., 2017), avoiding the need for host cells. However, the variety of proteins in microarrays is still significantly less than the spectrum of antigenic targets found in serum (Wang et al., 2020).

Peptide arrays focus on small protein segments instead of full-length proteins and are valuable in detailed epitope mapping within proteins that are already implicated in autoimmune diseases (Hecker et al., 2016). Peptide arrays can cover the entire human proteome, aiding in the discovery of new autoantigens, as shown in studies on multiple sclerosis (Zandian et al., 2017). However, peptide arrays have limitations, such as not capturing conformational epitopes and lacking post-translational modifications present in full-length proteins. Further, the high resolution provided by peptides comes with increased complexity, as multiple peptides are needed to fully cover a single protein.

Some methods such as MISPA and PepSeq which are based on protein or peptide arrays have been developed with significant improvements. Multiplexed In-Solution Protein Array (MISPA) is a quantitative platform that employs a solution-based protein library (Song et al., 2024). MISPA uses a full-length protein that allows the detection of conformational epitopes. The current MISPA configuration supports the analysis of up to 200 different antigens, but expanding the range is limited by the increased cost of preparing additional proteins. PepSeq is an *in vitro* platform for high multiplex proteomic assays using DNA-barcoding peptides, effective for detailed epitope analysis with minimal serum or plasma. It is a cost-effective, high-resolution tool for studying antibody epitopes and pathogen exposure (Henson et al., 2023). However, similar to other peptide-based serology assays, PepSeq cannot detect antibodies that rely on complex secondary, tertiary, and quaternary structures or post-translational modifications that do not present in unmodified linear peptides.

Having explored the wide-ranging capabilities of protein or peptide arrays and their improved techniques, along with their inherent advantages and limitations, we shall now turn our attention to display technologies that offer a unique approach to studying autoantibodies.

PhIP-Seq was first introduced to detect autoantibodies and identify autoantigens associated with patients suffering from paraneoplastic neurological syndromes (Larman et al., 2011). It leverages second-generation sequencing and nucleic acid synthesis technologies, providing the advantages of high throughput, improved sensitivity, and reproducibility (Vazquez et al., 2022). The key improvement in PhIP-Seq lies in its use of computer-designed and custom-built libraries. Unlike traditional phage display methods, PhIP-Seq libraries consist of sequences with defined lengths, overlaps, and known annotations. OLS is used in PhIP-Seq to generate libraries of oligonucleotides that encode peptide blocks spanning a library of protein sequences. OLS allows for the design of uniformly sized oligonucleotide libraries with specific codon usage to minimize bias. This enables the production of libraries consisting of thousands of proteins from hundreds of organisms in a matter of weeks. Compared to microarray technology, PhIP-Seq has advantages in terms of throughput and cost of analysis (Qi et al., 2022). PhIP-Seq eliminates the need to express recombinant proteins. Furthermore, the length of PhIP-Seq peptides is limited only by DNA synthesis chemistry, allowing for peptides of up to 90 amino acids in length (Mohan et al., 2018).

Molecular Indexing of Proteins by Self-Assembly (MIPSA) is a new technology that displays over 11,000 full-length proteins on ribosomes using a reconstituted cell-free system, each with a unique DNA barcode for parallel sequencing (Credle et al., 2022). The use of full-length proteins in MIPSA enables the detection of the complexity of conformational and discontinuous epitopes. MIPSA has been used to investigate autoreactive antibodies in severe SARS-CoV-2 infections (Credle et al., 2022). MIPSA’s ability to display full-length proteins with post-translational modifications and complex secondary structures complements the linear epitope mapping capabilities of PhIP-Seq, making MIPSA an ideal companion technology. Together, MIPSA and PhIP-Seq offer a synergistic approach to autoantibody profiling.

Rapid Extracellular Antigens Profiling (REAP) is also a new immunoproteomic technique that specializes in the detection of autoantibodies against the human exoproteome and secreted proteins (Wang et al., 2022). Utilizing yeast cells as eukaryotic expression hosts, REAP displays a panel of extracellular proteins, each genetically barcoded for precise identification via next-generation sequencing. REAP focuses on extracellular proteins, which are often complex and challenging to study with other methods. REAP has been used to identify autoantibodies in autoimmune diseases such as APS-1 and SLE (Wang et al., 2022), as well as in patients with severe SARS-CoV-2 infections (Wang et al., 2021), highlighting its utility in revealing the immune response against extracellular targets.

In summary, advancements in autoantibody profiling have been marked by the emergence of high-throughput techniques. Each technique, from the comprehensive protein interaction analysis offered by protein arrays to the detailed epitope mapping provided by peptide arrays, brings unique capabilities to the study of autoantibodies, MISPA and PepSeq have enhanced the array methods by linking DNA-barcoding and solution-based approaches. PhIP-Seq excels in the high-throughput identification of autoantigens, leveraging advanced sequencing and synthetic technologies to create extensive peptide libraries rapidly. MIPSA displays full-length proteins, capturing the conformational and post-translational complexity. Meanwhile, REAP specializes in profiling autoantibodies against the human exoproteome and secreted proteins, focusing on extracellular targets that are challenging for other methods. Collectively, these methods offer a robust toolkit for unraveling the complexities of autoimmune diseases, enabling the discovery of novel autoantigens, and advancing our understanding of immune responses.

## 3 Workflow of PhIP-Seq

**FIGURE 2:**
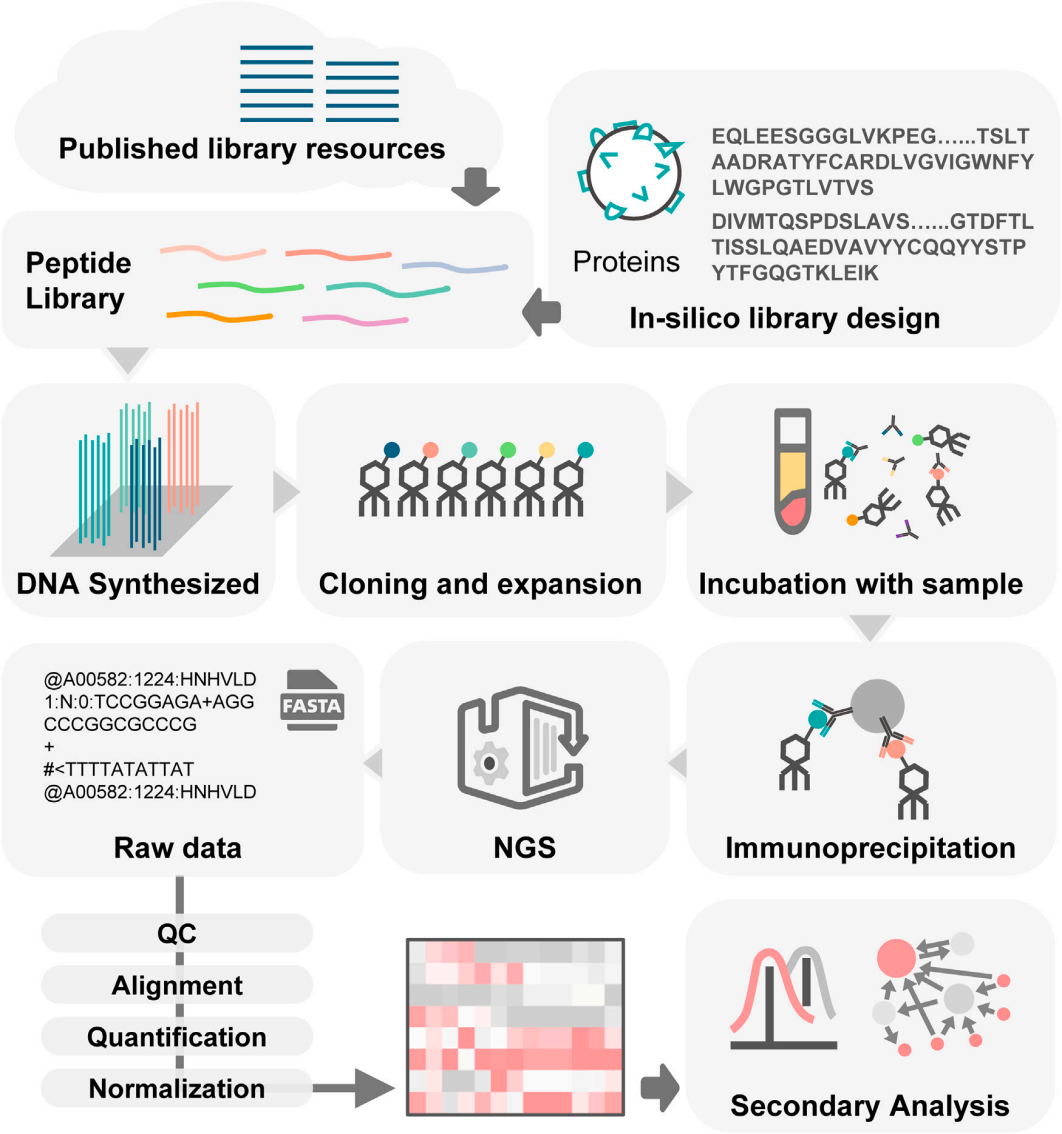
Workflow of PhIP-Seq. PhIP-Seq involves several key steps: (1) A peptide library is designed or downloaded. (2) The oligonucleotide library encoding the peptide sequences is synthesized. (3) Propagation of phage library. (4) Patient antibody-antigen interaction. (5) Conducting immunoprecipitation. (6) Creating DNA sequencing libraries followed by performing deep sequencing. (7) Data analysis and validation.

The process of PhIP-Seq involves several key phases (Figure 2): downloading or designing a peptide library, assembling the phage library, generating phage-antibody complexes, conducting immunoprecipitation, creating DNA sequencing libraries and performing deep sequencing, followed by analysis of the results.

### 3.1 PhIP-Seq library design

The process of PhIP-Seq can be initiated either by choosing peptide libraries from existing published libraries or creating custom-built libraries for specific experimental needs. The PhIP-Seq library design ensures the intact representation of all potential linear epitopes for a given set of proteins. Each epitope is typically 6–20 amino acids long (El-Manzalawy et al., 2008) and is fully contained within the displayed peptides. The design of the PhIP-Seq library mainly involves four steps: (1). Collecting target protein sequences; (2). Segmenting the protein sequences into peptides; (3). Adding forward and reverse PCR primers; (4). And finally, a rigorous scanning procedure to detect and eliminate any potential restriction sites. In the scanning procedure, synonymous mutations are introduced ensuring that the final open reading frames (ORFs) are free from rare codons and restriction sites that could hinder cloning efficiency. To facilitate these steps, a suite of bioinformatics tools has been developed, each offering unique features and advantages that significantly expedite the library design process and enhance the overall reliability of the PhIP-Seq methodology.

Pepsyn is the commonly used tool for PhIP-Seq library design. It is a Python-based open-source software package. Initially, representative protein sequences are selected by Pepsyn using CD-HIT, a tool that clusters and filters sequences based on similarity and redundancy. Then it tiles the sequences to obtain overlapping peptides using a sliding window approach (Mohan et al., 2018). In 2022, the open-source tool BIPS (BuildPhIPSeqLibrary) was developed. This tool enables the complete representation of multiple variants in an unambiguous manner while allowing peptide identification without the need for full-length sequencing (Leviatan et al., 2022a). BIPS improves traditional library design methods by efficiently handling large and complex peptide libraries with numerous similar sequences. It overcomes the limitations of partial peptide sequencing and the high costs and errors associated with full oligo sequencing or external barcodes. By integrating internal barcodes within the oligos, BIPS ensures accurate peptide identification without the need for lengthy or expensive sequencing processes, thereby enhancing the overall efficiency and reliability of PhIP-Seq technology. Furthermore, it is crucial to display antigenic epitopes selectively in order to develop efficient peptide libraries. With the advancements in machine learning, several machine-learning methods are now available for antigenic epitope (B-cell epitope) prediction (Saha and Raghava, 2006; Lian et al., 2015; Liu et al., 2020). Recently, a PhIP-Seq library design algorithm called Dolphyn which is based on the Random Forest Epitope Predictor was developed. It can compress the size of a peptide library by 78% compared to traditional tiling while increasing the number of antibody-reactive peptides from 10% to 31% (Liebhoff et al., 2024). Currently, there is a relatively limited variety of PhIP-Seq library design software available to users. Apart from the three mentioned open-source tools, most software options require payment or are limited to in-house use, making it more difficult for outside researchers to access relevant resources.

### 3.2 PhIP-seq protocol methods

As PhIP-Seq gained more attention in the field of immunology, several studies were published with comprehensive protocols for PhIP-Seq (Mohan et al., 2018; Shrock et al., 2022; Vazquez et al., 2022; Raghavan et al., 2023). Despite the variations in the aims, the core laboratory procedures remain consistent across these studies. The process typically begins with the meticulous construction of a diverse phage screening library, which acts as a platform for presenting a wide range of peptides. Subsequently, phage-antibody complexes are formed followed by immunoprecipitation to enrich antigen-specific antibodies. The final step is the library preparation for next-generation sequencing and execution of NGS. These fundamental steps form the foundation of the PhIP-Seq methodology, enabling systematic profiling of the humoral immune response and identification of antigen-antibody interactions with high resolution and high throughput.

The original experimental steps of PhIP-Seq, as described in the seminal work by Larman et al. (2011), have been further developed and refined. Based on their previous work, in which they meticulously described the experimental procedures used in PhIP-Seq, they published a comprehensive protocol in 2018. The aim was to offer a more user-friendly and applicable guide for the scientific community (Mohan et al., 2018). Although PhIP-Seq is an efficient technology that allows for multiplexing, it still requires a substantial amount of labor. To address these limitations, a scaled PhIP-Seq protocol has been developed (Vazquez et al., 2022). This advancement introduced a vacuum-based system that can process 600–800 samples. It greatly reduces differences between batches and the risk of contamination. This high-throughput extension of PhIP-Seq not only makes it more accessible by removing the need for robots but also allows for the inclusion of larger control groups which is crucial for identifying disease-specific autoantibodies.

The inclusion of videos in the VirScan protocol (Shrock et al., 2022) enhances its practical application by making procedural details easier for researchers to understand. This strategic enhancement improves the technique’s usability and ensures that researchers can visually comprehend the process. This approach to protocol dissemination is particularly instrumental in standardizing experimental techniques and promoting reproducibility across laboratories.

Furthermore, it is important to note that the T7 phage display vector is commonly used in PhIP-Seq experiments. The T7 phage has a smaller viral particle size, around 60 nm in diameter, and can accommodate oligonucleotides up to 40 kb in length. T7 phage can display large protein fragments of up to 1,000 amino acids and is modified to express different amounts of 10 B *versus* 10 A proteins, showing peptide sequences in 1–415 copies. In contrast, the M13 phage typically displays short peptides in thousands of copies due to steric hindrance with larger proteins, and the T4 phage utilizes the non-essential coat proteins SOC and HOC for displaying foreign peptides or proteins (Tan et al., 2016). Compared to other vectors, the T7 phage exhibits a rapid replication cycle, robust stability in unfavorable conditions, an enhanced capacity for displaying larger proteins, and superior cloning efficiency (Jaroszewicz et al., 2022). These characteristics position T7 as a preferred vector for probing and elucidating pathogen-host interactions in PhIP-Seq studies.

### 3.3 Analysis of PhIP-Seq data

Generating phage libraries is a well-established technique. However, deciphering the resulting data is still complex. The main challenge is the large amount of sequence information generated, which requires advanced bioinformatics tools and expertise for effective analysis. Consequently, transforming the raw data into actionable insights requires a multidisciplinary approach and significant effort.

The PhIP-Seq data analysis process involves a systematic pipeline to handle raw data, converting complex sequencing information into interpretable biological data. It also includes various subsequent data analysis methods. The typical processing of raw data consists of data filtration, demultiplexing, alignment to the reference sequence, and ultimately quantification and normalization. In the secondary analysis, the focus lies on the enrichment analysis of phage peptides.

Data filtration is a crucial step in improving data quality and vital for guaranteeing the accuracy and reliability of later analyses. Initially, raw data undergo primary quality control to remove low-quality reads with ambiguous base calls or insufficient depth. Adapter sequences are then trimmed to prevent interference in subsequent analyses. Additionally, reads with high homopolymer or repetitive sequences are filtered out to minimize biases. Data filtration is essential for ensuring the accuracy and reliability of downstream analyses.

Following data filtration, demultiplexing separates the combined sequencing reads and assigns them to their corresponding samples using molecular identifiers. Subsequently, the reads are aligned to the reference proteome using various established bioinformatics tools such as Bowtie (Langmead et al., 2009), GSNAP (Wu and Nacu, 2010), and RAPSearch2 (Zhao et al., 2012).

In the quantification and normalization process, the read count for each specific peptide tile is determined for every sample. This is followed by a normalization procedure to adjust the read counts and overcome the bias introduced by varying sequencing depths. Normalizing all sequencing data to a consistent level is necessary before further analysis. In RNA-Seq data analysis, normalization is essential to address technical variations such as sequencing depth and gene length. Common methods include TPM (Transcripts Per Million), FPKM (Fragments Per Kilobase of exon per Million reads), and edgeR’s Trimmed Mean of M-values (TMM). Similarly, PhIP-Seq also utilizes NGS platforms to generate a large number of reads for each sample. In some studies, PhIP-Seq data is normalized by converting raw read counts to percentages of total reads per sample (Mandel-Brehm et al., 2019; Vazquez et al., 2020; Vazquez et al., 2022). For instance, The Read Counts Per Million (RPM) method is used for normalizing PhIP-Seq data, which involves normalizing the read count of each peptide by the total read counts of the sample and then multiplying by one million (Yuan et al., 2018). Additionally, a study that has evaluated the applicability of edgeR’s normalization and analysis methods for PhIP-Seq demonstrates that edgeR also can be used for normalizing and analyzing PhIP-Seq data (Chen et al., 2022b). Moreover, Phip-flow offers the CPM (Counts Per Million) method for normalizing PhIP-Seq data (Galloway et al., 2023).

In the analytical segment of PhIP-Seq data processing, some bioinformatics tools are employed to assist in the process, as detailed in Table 2. A unified or standard PhIP-Seq data processing pipeline is crucial for integrating, standardizing, and reliably analyzing experimental data. Jared G. Galloway et al. developed Phippery, a software that can perform integrated processing of PhIP-Seq raw data (Galloway et al., 2023). This software enables data cleaning, quality control, comparison, and data normalization. By default, alignment is performed with Bowtie2, peptide counts are collated with SAMtools, and normalization is performed via edgeR. Additionally, Phippery includes an interactive visualization module that allows for flexible visualization as heatmaps.

**TABLE 2:** Tools for PhIP-Seq.

Tools	Function	Description	Application	Date	Sources
Pepsyn	Library design	Using representative protein sequences to generate overlapping peptides, the length of the peptides and the overlap can be set	A smaller antigenic spaces or lower precision requirements	2016	https://github.com/lasersonlab/pepsyn
BIPS Leviatan et al. (2022a)	Library design	Convert the protein sequence into peptides, and create internal barcodes through synonymous mutations to ensure the uniqueness and recognizability of each peptide	Saving on the costs of sequencing and maintaining the accuracy of oligonucleotide reads identification	2022	https://github.com/kalkairis/BuildPhIPSeqLibrary
Dolphyn Liebhoff et al. (2024)	Library design	Predict epitope peptides using machine learning and condense the antigen space by combining epitope peptides	Efficient encoding of large antigenic space	2024	https://github.com/kepsi/Dolphyn
PairSeq	Data analysis	An R library that simplifies the process of antigen identification from phage display sequencing data	Distinguishing true antigens from off-targets	2017	https://github.com/cmbartley/PairSeq
Z-score Yuan et al. (2018)	Data analysis	A statistical model to quantify antibody-dependent changes in phage clone abundance	Determining the enrichment score	2018	https://github.com/LarmanLab/PhIP-Seq-Analyzer
BEER Chen et al. (2022a)	Data analysis	A Bayesian framework for identifying reactive peptides	Quantify peptide reactivity in PhIP-Seq	2022	https://bioconductor.org/packages/release/bioc/html/beer.html
AVARDA Monaco et al. (2022)	Data analysis	A multi-module software package for analyzing VirScan datasets	VirScan data	2022	https://github.com/drmonaco/AVARDA
Phippery Galloway et al. (2023)	Pipeline	Provide end-to-end automation in reproducible workflows for PhIP-Seq data, offering interfaces for tasks such as count normalization, enrichment calculation, multidimensional scaling, and data visualization	PhIP-Seq raw data processing and secondary analysis	2023	https://github.com/matsengrp/phippery

In downstream analysis, the main objective is to identify the presence of antibody-bound peptides. Enrichment analysis plays an important role in both qualitatively and quantitatively determining peptide binding. This process involves assessing the relative abundance of peptides that are bound by antibodies. Significantly enriched peptides, referred to as “hits,” are identified using two common methods: statistical testing and scoring metrics.

The statistical approach hinges on the calculation of *p*-values, providing a quantitative measure of the probability that the observed enrichment of a peptide could occur by chance. In PhIP-Seq data, Generalized Poisson (GP) models are often used to estimate *p*-values associated with peptide enrichment. The Poisson distribution is based on the assumption that events are independent and occur randomly over a fixed interval of time or space. In the context of PhIP-Seq, it is assumed that the binding of peptides is independent. Initially, the clonal abundance distribution of the starting library is determined by sequencing. Subsequently, the observed abundance distribution of the corresponding peptides in an enriched library is fitted to a GP distribution for each abundance interval in the starting library. Model parameters are then estimated for each interval, allowing for the formulation of an expected GP distribution for all peptides using the regressed model parameters. A peptide is considered a “hit” if the *p*-value falls below a predetermined threshold. For instance, in pioneering studies of PhIP-Seq, several statistical distribution families were assessed for their ability to appropriately model the PhIP-Seq enrichment data. It was determined that the two-parameter generalized Poisson distribution was the best choice (Larman et al., 2011). This model assumes that the data may exhibit overdispersion or underdispersion relative to the standard Poisson distribution, introducing an additional parameter to account for this dispersion, making it more flexible for fitting real-world data where the variance is not equal to the mean. In another study, the authors discovered that a zero-inflated generalized Poisson distribution could adeptly fit the frequency of each peptide. They accomplished this by fitting the zero-inflated generalized Poisson model to the distribution of output counts and regressing the parameters at a particular input read count (Xu et al., 2016). Zero-inflated generalized Poisson distribution is based on the assumption that there are more zero counts than expected in a typical Poisson distribution, combining a point mass at zero with a generalized Poisson distribution for non-zero counts. Moreover, the Larman group proposed a variation of the generalized Poisson model, introducing a Gamma-Poisson mixture model to fit the data better. This new approach was implemented in phip-stat (https://github.com/lasersonlab/phip-stat) and Phippery (Galloway et al., 2023). Apart from GP models, other methods can be used to identify “hits.” For example, one method has been incorporated into BEER (Chen et al., 2022a) and is specifically designed for analyzing PhIP-Seq data. This method uses a negative binomial distribution to fit the data and a Bayesian hierarchical model to compute the posterior probabilities of peptide enrichment. BEER also offers peptide reactivity inference using the edgeR package, with faster speed but slightly lower sensitivity for weakly reactive peptides compared to the Bayesian approach.

Scoring metrics evaluate the degree of peptide enrichment through alternative quantitative criteria. These metrics encompass fold-changes in peptide abundance, or z-score (Yuan et al., 2018). A peptide may be designated as a “hit” if it surpasses a certain threshold within this scoring system. A z-score represents the standard deviation of an observation (e.g., peptide abundance) from the mean of a reference distribution (e.g., background peptide abundance in negative controls). Peptides with z-scores above a certain threshold are considered enriched (Eshleman et al., 2019; Vazquez et al., 2022; Raghavan et al., 2023). The fold change in the abundance of a peptide between experimental and control conditions can also be used to infer enrichment (Vazquez et al., 2022). Moreover, in a study, identifying such hits depended on a comprehensive evaluation that included several important metrics (Monaco et al., 2021). Specifically, for a peptide to be classified as a hit, it had to meet stringent criteria across three different parameters: counts, *p*-value, and fold change (FC).

In the downstream analysis process, various methods and tools have been developed to support and streamline different aspects of PhIP-seq data analysis, including PairSeq, BEER (Chen et al., 2022a) and AVARDA (Monaco et al., 2022). PairSeq is an R Package that simplifies the process of antigen identification from phage display sequencing data and identifies enriched peptides that share epitopes. It addresses the challenge of distinguishing true antigens from off-targets. PairSeq extracts and ranks peptides that are both enriched and overlapping, improving the accuracy of antigen selection. BEER is a software package specifically developed for the quantification of peptide reactivity from PhIP-Seq experiments. AVARDA offers a systematic and probabilistic framework for analyzing highly multiplexed antiviral antibody epitope reactivity data. It provides adjusted *p*-values for virus exposure, the breadth of antibody responses, and the relationships between reactive peptides, thereby improving the accuracy and comprehensiveness of antiviral antibody response analysis.

## 4 Application of PhIP-Seq

In Table 3, we present a compilation of PhIP-Seq libraries that have been published in recent years. These libraries not only offer significant insights into health and disease but also lay the groundwork for further discoveries. These comprehensive libraries encompass peptides belonging to a diverse range of biological entities including human proteins (Larman et al., 2011; Xu et al., 2016; O'Donovan et al., 2020), common viruses (Xu et al., 2015), allergens (Monaco et al., 2021), human microbiota (Vogl et al., 2021), non-viral environmental and microbial toxins and virulence factors (Angkeow et al., 2022). Moreover, these highly versatile, peptide sequences are readily accessible for oligonucleotide library synthesis through publicly available resources.

**TABLE 3:** Details of published PhIP-Seq libraries.

Library	Description	Size	Length	Overlap	Date
HuScan Larman et al. (2011)	Human peptidome library from all 24,239 open reading frames of Human Genome version 35.1	413,611	36aa	7aa	2011
Human PhIP-Seq Library v2 O'Donovan et al. (2020)	Human proteome library, from all human sequences in the NCBI protein database, including all splicing isoforms and computationally predicted coding regions	731,724	49aa	25aa	2020
VirScan Xu et al. (2015)	The human virome library, comprising all protein sequences from over 1,000 strains of the 206 human-tropic viruses in the UniProt	93,904	56aa	28aa	2015
T7-HCoV-56-mer library Shrock et al. (2020)	Including the open reading frames (ORFs) expressed by six human coronaviruses (HCoVs) and three bat coronaviruses closely related to SARS-CoV-2	Unknown	56aa	28aa	2020
SARS CoV-2 triple-alanine scanning library Shrock et al. (2020)	A triple alanine scanning mutant of the 56-mer peptide tiling in the SARS-CoV-2 proteome	Unknown	56aa	28aa	2020
SARS-CoV-2 20-mer library Shrock et al. (2020)	From the encoding of the SARS-CoV-2 proteome	Unknown	20aa	5aa	2020
HuCoV Zamecnik et al. (2020)	From the genomes of 9 HuCoVs	3,670	38aa	19aa	2020
SARS-CoV-2 Zamecnik et al. (2020)	From the encoding of the SARS-CoV-2 proteome	534	38aa	19aa	2020
hCoV and aCoV antigen library Klompus et al. (2021)	Covering all 7 hCoVs and 49 aCoVs originating from diverse hosts including bats, rodents, domestic animals, and birds	12,924	64aa	20aa	2021
Phageome Angkeow et al. (2020)	Based on sequencing of environmental phages and large-scale metagenomic sequencing of virus-like particles isolated from stool samples from IBD patients and their non-IBD household contacts	Unknown	Unknown	Unknown	2020
Spike Phage-DMS Garrett et al. (2021)	High-resolution Spike-specific deep mutational scanning phage display library	24,820	31aa	30aa	2021
AnelloScan Venkataraman et al. (2022)	Represent 829 anellovirus genomes	32,960	56aa	28aa	2022
Mouse peptide library Rasquinha et al. (2022)	49,734 unique protein sequences from the mouse proteome from the UniProt database	419,915	56aa	Unknown	2022
*Mus musculus* proteome-wide library Rackaityte et al. (2023)	76,217 mouse protein sequences from the GRCm38.p5 *Mus musculus* genome	482,672	62aa	19aa	2023
T7 AllerScan Monaco et al. (2021)	1847 protein sequences from the Allergome database	19,332	56aa	28aa	2021
Microbiota Peptide Library Vogl et al. (2021)	Proteins of intestinal pathogens identified through metagenomic sequencing and human self-antigens from the VFDB and IEDB databases, comprising approximately 28,000 proteins	244,000	64aa	20aa	2021
Food and environmental antigens library Leviatan et al. (2022b)	The total number of 7,541 proteins encompasses 1,434 proteins from the IEDB and 6,107 proteins from allergen databases	58,233	54aa	20aa	2022
ToxScan Angkeow et al. (2022)	The 14,430 diverse proteins with “toxin” or “virulence factor” included as keywords in the UniProt database	95,601	56aa	28aa	2022
Falciparome library Raghavan et al. (2023)	From 8,980 protein sequences, including all known protein sequences from 2 falciparum References strains and diverse variant sequences of key antigens	238,068	62aa	37aa	2023
S. mansoni library Woellner-Santos et al. (2024)	11,641 known proteins from all S. mansoni life-cycle stages	117,641	58aa	7aa	2024

### 4.1 Autoantibody discovery in human populations

A human peptide library uniformly expresses the entire human proteome in an array of overlapping short peptides (peptidome) displayed on phages. This unique resource serves as a valuable tool for research purposes, especially in the study of autoimmune diseases. The loss of tolerance to self-antigens in humans leads to diseases including type I diabetes, rheumatoid arthritis, and multiple sclerosis which are also called autoimmune diseases. Therefore, the study of autoantibodies resulting in the autoimmune processes is beneficial in the understanding of disease causation as well as in developing more accurate diagnostic tests (Larman et al., 2011). PhIP-Seq technology aids in the efficient identification of autoantibodies by utilizing phages to display human peptides that bind to the antibodies in patient samples. This approach has played a crucial role in uncovering disease-specific autoantibody profiles and assessing autoantibody reactivity in healthy individuals (Jaycox et al., 2024). Furthermore, PhIP-Seq has greatly contributed to our understanding of the mechanisms underlying autoimmune diseases and some neurological diseases (Prüss, 2021).

#### 4.1.1 Human peptide libraries

Initially, Larman et al. (2011) created the first synthetic representation of the entire human proteome as a peptidome library called HuScan™ on the surface of T7 phage when they proposed the idea of PhIP-Seq technology. HuScan™ includes 413,611 36-mer peptides with seven residues overlapping between consecutive peptides. These peptides were based on all 24,239 open reading frames of the human genome version 35.1. Larman et al. (2013) used this library to identify autoantibodies in patients with paraneoplastic neurological syndrome. In 2013, HuScan™ was employed to conduct large-scale PhIP-Seq screening on individuals with various autoimmune diseases such as multiple sclerosis, type 1 diabetes, and rheumatoid arthritis. The database that was generated from peptide-antibody interactions identifies the distinct autoantibody fingerprint of each person including common specificities observed in the general population and those linked to particular diseases.

In 2020, the new Human PhIP-Seq Library v2 was designed, significantly broadening the scope of this field. This updated library encompasses all annotated human protein sequences in the NCBI protein database, including all known splice isoforms and predicted coding regions. This library contains 7,31,724 sequences, significantly expanding the breadth of potential autoantigen targets and enhancing the capability to detect autoantibodies in comparison to HuScan™ (O’Donovan et al., 2020). Moreover, utilizing this advanced library, high-resolution autoantibody epitope profiles were created for patients with anti-Yo and anti-Hu syndromes which are two common paraneoplastic neurological disorders. The results of this research further emphasize the effective usage of phage immunoprecipitation sequencing in fundamental and clinical research as well as in gaining deeper insights into the antigenic targets and factors triggering paraneoplastic neurological disorders (O’Donovan et al., 2020). Furthermore, an “autoreactome”, has been constructed for 78 healthy individuals using the Human PhIP-Seq Library v2. This study focuses on the comprehensive exploration of the regulatory mechanisms of autoantibody profiles at the proteomic level in both healthy and disease states providing valuable insights into the understanding of human immune regulation (Bodansky et al., 2023b). The PTM-modified PhIP-Seq library developed by Román-Meléndez et al. (2021) in 2021 addressed a critical gap that was present in previous PhIP-Seq approaches. This library incorporated citrullinated peptides through enzymatic modification with PAD2 and PAD4 overcoming the previous limitation of not being able to include post-translational modifications (PTMs). It consists of approximately 250,000 overlapping 90 amino acid peptide tiles spanning the human proteome. This advancement allowed for a more accurate and comprehensive analysis of autoantibody reactivities that were directed against modified proteins.

The human PhIP-Seq library is a powerful tool for discovering autoantibodies and its applications are widespread in sero-epidemiological studies, autoimmune disease research, and the identification of novel antigens and epitopes. Human peptide libraries are also valuable in assessing seroprevalence, understanding disease etiology, and monitoring vaccine responses.

#### 4.1.2 Autoimmune disease

Through high-throughput antibody profiling, PhIP-Seq has revealed the specificity and diversity of autoantibodies in various autoimmune diseases. For instance, in a study on primary membranous nephropathy (MN), PhIP-Seq characterized the circulating antibody libraries in patients, although no significant differences in autoantigen specificity were found between MN patients, chronic kidney disease (CKD) patients, and healthy controls (Cantarelli et al., 2020). In addition, PhIP-Seq successfully generated detailed profiles of autoantibody epitopes and identified 17 distinct genes that encode ILD-rich autoantigens in studies on idiopathic pulmonary fibrosis (IPF), hypersensitivity pneumonitis (HP), and connective tissue disease-associated ILD (CTD-ILD) (Upadhyay et al., 2023). The application of PhIP-Seq in neurological autoimmune diseases such as multiple sclerosis (MS) and myasthenia gravis (MG) has provided information crucial in uncovering the immunologic basis of these diseases (Hosono et al., 2023). Recently, a distinctive autoantibody signature that can predict the onset of MS in a subset of patients has been identified through PhIP-Seq. Serum samples from over 10 million individuals were analyzed in this study, finding a unique cluster of autoantibodies in about 10% of MS patients. This signature, which targets a common motif similar to many human pathogens, was present years before the onset of MS symptoms and was associated with higher levels of serum neurofilament light (sNfL). These findings suggest a potential role for these autoantibodies as early biomarkers for MS (Zamecnik et al., 2024).

Furthermore, PhIP-Seq has been used to identify disease biomarkers. For instance, cavin-4 IgG has been recognized as a serological indicator in immune-mediated rippling muscle disease (Dubey et al., 2022). Additionally, a new autoantigen, the transcription factor Sp4, has been identified as being associated with an increased risk of cancer in patients with dermatomyositis (DM) (Hosono et al., 2023). In addition to biomarker discovery, PhIP-seq has also been utilized in aiding the diagnosis of diseases. A machine learning classifier for diagnosing autoimmune hepatitis (AIH) has been developed using PhIP-seq data (Klepper et al., 2023).

In addition, the field has made further advancements by utilizing a citrullinated phage-displayed human peptidome library. This tool has been instrumental in elucidating the specificities of autoantibodies associated with rheumatoid arthritis (Román-Meléndez et al., 2021). This innovative approach employed PhIP-Seq to profile anti-citrullinated protein antibody (ACPA) reactivities. By incorporating both unmodified and peptidylarginine deiminase (PAD)-modified phage display libraries, this study successfully identified antibodies targeting citrulline-dependent epitopes.

#### 4.1.3 Neurological disease

Paraneoplastic neurological syndromes (PNS) are rare yet refractory disorders that occur as remote effects of cancer, characterized by immune-mediated pathogenesis (Graus et al., 2021). The underlying cause of these disorders involves immune responses mediated by cells that are specific to antibodies or autoantigens. These antibodies or autoantigens target onconeural antigens that are found in tumors. PhIP-Seq has shown promising results in detecting new autoantibody biomarkers in PNS. In 2019, the Human PhIP-Seq Library v2 was used to discover antibodies to kelch-like protein 11 (KLHL11) in the cerebrospinal fluid and serum of 13 patients with seminoma-associated paraneoplastic encephalitis (Mandel-Brehm et al., 2019). In 2021, HuScan have been employed to identify autoantibodies in paraneoplastic central nervous system (CNS) disease. TRIM46 was then confirmed as the autoantigen by TRIM46 cell-based assay and colocalization on mouse brain sections. This finding provides compelling evidence supporting the potential utility of TRIM46-IgG as a promising biomarker for diagnosing and monitoring this disease (Valencia-Sanchez et al., 2022). In 2022, research reported that βIV-Spectrin autoantibodies are specific biomarkers for paraneoplastic neuropathy (Bartley et al., 2022a). In 2023, a novel biomarker, SKOR2 IgG, for PNS was identified using PhIP-Seq (Rezk et al., 2023). Furthermore, the application of PhIP-Seq for detecting ZSCAN1 autoantibodies in patients with Rapid-onset Obesity with Hypothalamic Dysfunction, Hypoventilation, and Autonomic Dysregulation (ROHHAD) provided further evidence to support the hypothesis that ROHHAD syndrome is a pediatric peripheral neurological disorder with neoplastic implications (Mandel-Brehm et al., 2022).

#### 4.1.4 Viral infection-related autoantibodies

PhIP-Seq human peptide libraries have also played a crucial role in studying viral infection-related autoimmunity. For instance, HuScan™ has been used to investigate the level of autoimmune antibodies in COVID-19 patients with different severities (Nasarallah et al., 2022). Autoimmunity has been proposed as one potential mechanism driving long COVID. HuScan™ also facilitated the exploration of COVID-19 sequelae, revealing significant differences in autoreactivity between those infected with SARS-CoV-2 and pre-COVID controls. This differential enrichment, composed of peptides from diverse intracellular proteins, suggests that the observed autoreactivity may result from cross-reactivity with SARS-CoV-2-directed antibodies in those exposed to the virus (Bodansky et al., 2023a). Similarly, The Human PhIP-Seq Library v2 has been used to identify candidate autoantigens in COVID-19 patients with neurological symptoms (Song et al., 2021), and identify autoantibodies targeting neural proteins in Human Immunodeficiency Virus (HIV) patients with steroid-responsive meningoencephalitis (Bartley et al., 2022b).

The studies mentioned above emphasize the critical role of PhIP-Seq human peptide libraries in investigating various autoimmune diseases and highlight the importance of identifying autoantigens to enhance our understanding of disease mechanisms.

### 4.2 Anti-microbial antibody discovery

#### 4.2.1 Human virus

Following the significant progress in autoimmune antibody identification using the human proteomic PhIP-Seq library, Larman et al. designed VirScan, a T7 phage-displayed human virome library in 2015. VirScan encompasses 93,904 peptides, each consisting of 56 amino acid residues. These peptides were based on the protein sequences of all 206 humanophilic viruses found in the UniProt database (Xu et al., 2015). VirScan was used to analyze antibody-virus peptide interactions in a study involving 569 humans from four continents. On average, antibodies against 10 distinct viral species were identified per individual, with at least two individuals demonstrating reactivity to as many as 84 viral species. It was also observed that the population exhibited a strong antibody response to a common epitope conserved by each virus (Xu et al., 2015). In summary, VirScan offers an invaluable tool for investigating the interplay between the immune system and the virome, providing a high-dimensional view of global immunity against human viruses. Furthermore, VirScan-based HIV antibody profiles can be utilized for analysis of the comprehensive specificity of antibody responses throughout all stages of HIV infection (Eshleman et al., 2019). VirScan has been used to demonstrate the connection between enterovirus infections and acute flaccid myelitis (AFM) as well as type I diabetes, thereby providing evidence for a causal role of non-polio enteroviruses in AFM (Schubert et al., 2019; Monaco et al., 2022). VirScan has also been employed for the characterization of placental transfer of viral antibodies (Pou et al., 2019). Additionally, VirScan has shown great potential for the surveillance of antibodies against human viruses in bats. It has been used to analyze antiviral antibodies against over 200 human viruses in serum samples from *Pteropus alecto* and *Eonycteris spelaea*. VirScan offers a significant alternative for future wildlife surveillance efforts. It holds great promise for expanding human biomedical technologies for additional human disease surveillance applications in wildlife hosts (Ruhs et al., 2023). In the field of cancer research, VirScan has been shown to have the potential to predict the response to immune checkpoint blockers (ICBs) in NSCLC patients by assessing viral infections (Dall’Olio et al., 2022). In another study on hepatocellular carcinoma, VirScan was able to identify a range of viral strains, including viral hepatitis and non-hepatitis viruses, that exhibited positive or negative associations with hepatocellular carcinoma. This may potentially reflect a causal relationship between non-pathogenic viral infections and tumorigenesis (Do et al., 2023).

In 2022, the AnelloScan library was created using open reading frames from over 800 human anelloviruses. This study provides more insights into the adaptive immune responses to Anelloviruses which are an important part of the human commensal virome (Venkataraman et al., 2022).

#### 4.2.2 Against SARS-CoV-2

In response to the global COVID-19 pandemic, targeted research and rapid response were crucial. As an effective tool for accurately identifying and analyzing pathogens, the PhIP-Seq viral library played a major role in these research efforts. Combined with virus-specific libraries, PhIP-Seq technology significantly enhanced the sensitivity and specificity of virus detection, thereby playing a pivotal role in outbreak surveillance, viral lineage tracing, vaccine development, and other pertinent domains.

Viral libraries such as the T7-HCoV-56-mer library, SARS-CoV-2 20-mer library, and SARS-CoV-2 triple-alanine scanning library have been developed for the detection of viral exposure history and identification of SARS-CoV-2 epitopes. These libraries facilitated the identification of private and public antibody epitopes, SARS-CoV-2 specific epitopes as well as those that cross-react with common-cold coronaviruses (Shrock et al., 2020). Furthermore, these viral libraries have been extensively utilized in various other research applications. The T7-HCoV-56-mer library was used to analyze COVID-19 convalescent plasma, revealing a correlation between antibody responses to endemic coronaviruses and the functionality of the plasma, which could have implications for understanding cross-reactivity with SARS-CoV-2 (Morgenlander et al., 2021). The T7-HCoV-56-mer library was also used to investigate the influence of prior infection with seasonal CoVs on the breadth and magnitude of anti-SARS-CoV-2 antibody responses. This study found a strong positive association between the magnitudes of anti-spike HCoV and anti-SARS-CoV-2 spike responses in cancer patients, but only a weak association in non-cancer patients, suggesting that prior infection with HCoVs might help limit SARS-CoV-2 infection and COVID-19 disease severity (Lidenge et al., 2024). Additionally, the T7-HCoV-56-mer library was used to assess the antibody response in vaccinated kidney transplant recipients (Karaba et al., 2023) and it also played a crucial role in the development of COVID-19 vaccines (Yalcin et al., 2023).

The HuCoV library contains 3,670 38-mer peptides covering nine human coronavirus genomes. This library was utilized to identify nine candidate antigens for SARS-CoV-2 within the serum of COVID-19 patients (Zamecnik et al., 2020). In a comprehensive study, this library was used alongside multi-omic analyses to gain deeper insights into the immune responses of COVID-19 patients. By integrating data from serology, proteomics, metabolomics, and transcriptomics, the study performed a systems-level analysis of the host-virus interactions and the dynamics of the immune response during infection (Diray-Arce et al., 2023).

Moreover, the Spike Phage-DMS library (Garrett et al., 2021), a high-resolution, deep mutation-scanning phage display library of SARS-CoV-2 spike proteins was also developed. It contains 24,820 31-mer peptides that cover the spike protein with 1-amino acid length increments. This study revealed that the SARS-CoV-2 mRNA vaccine induced more antigen-binding epitopes than post-infection, suggesting that the protective effect may be related to the pathway of exposure to the Spike antigen (Garrett et al., 2022).

Building upon the success of existing viral libraries, a comprehensive library that includes all seven human coronaviruses (hCoVs) and 49 animal coronaviruses (aCoVs) represented by 12,924 peptides was designed. The creation of regularly updated antigen libraries representing the animal coronavirome could provide a foundation for a serological assay capable of identifying infected individuals following future zoonotic transmission events (Klompus et al., 2021).

Also, a random peptide library in combination with PhIP-Seq technology has been successfully utilized to identify epitopes of SARS-CoV-2. This was demonstrated by the discovery of a high-affinity peptide called Spep-1 (Chen M. et al., 2022) that exhibits a strong binding capacity to the spike trimer protein, with specific amino acids within the S2 subunit mediating key interactions. The high affinity and specificity of this peptide highlight the potential of random peptide libraries in the development of diagnostic tools and therapeutic agents for COVID-19.

Furthermore, the combined use of the PhIP-Seq human proteome library and viral proteome library can successfully detect changes in autoantibodies following viral infection and the presence of pathogenesis-related autoantibodies (Rasquinha et al., 2022; Hawes et al., 2023) offering a potential basis for disease treatment.

#### 4.2.3 Food and environment

In 2021, AllerScan, a programmable phage display library, was developed for analyzing the reactivities of both IgE and IgG antibodies in individuals with food allergies. This library is based on 1,847 protein sequences retrieved from the Allergome database. The AllerScan contains 19,331 56-mer peptides and each peptide exhibits an overlap of 28 amino acid residues at both ends of its preceding and following polypeptide (Monaco et al., 2021).

In 2022, a comprehensive antigen library was constructed, encompassing a wide array of food and environmental proteins. This PhIP-seq peptide library comprises 58,233 peptides derived from 7,541 proteins obtained from multiple databases including WHO/IUIS, Allergome, AllergenOnline, SDAP, AllFam, and IEDB. It can be useful in analyzing human serum antibody responses to various food and environmental proteins. Thereby enhancing our understanding of the interplay between dietary antigens and the immune system. Such insights can potentially aid in the development of dietary recommendations and therapeutic interventions for immune-related conditions (Leviatan et al., 2022b).

Thomas Vogl et al. compiled 28,000 proteins from gut metagenomics sequencing, the Virulence Factor Database (VFDB), and the Immune Epitope Database (IEDB). Using this data, they created a 64-mer PhIP-Seq library, representing the microbiota, consisting of 2,44,000 peptides, each 64 amino acids in length. They assessed the functional serum Ig-epitope profiles in 997 individuals using this library and identified numerous public and private responses against gut microbiota antigens (Vogl et al., 2021). Building on this work, Thomas Vogl and his team explored the immunological landscape of myalgic encephalomyelitis/chronic fatigue syndrome (ME/CFS) using the microbiota peptide library. They identified a significant overrepresentation of antibodies against *Lachnospiraceae* bacteria flagellins in individuals with severe ME/CFS. This discovery, combined with machine learning analysis, highlights the involvement of the gut microbiota-immune axis in ME/CFS and suggests a potential diagnostic avenue (Vogl et al., 2022).

By combining the food and environmental library (Leviatan et al., 2022b) and microbiota peptide library (Vogl et al., 2021), a pivotal study on inflammatory bowel disease (IBD), was conducted. PhIP-seq was applied to scrutinize antibody response profiles in the serum of 497 IBD patients. This research identified 373 distinct antibody responses, with 55% specific to Crohn’s disease, 28% to ulcerative colitis, and 17% shared between both conditions. Significantly, antibody reactions to bacterial flagellin proteins predominated in Crohn’s disease and correlated with disease complications, independent of fecal microbiota composition. These findings underscore the heterogeneity of IBD and the potential of antibody profiling as a diagnostic and therapeutic tool (Bourgonje et al., 2023). In addition, the researchers discovered associations between phenotypic factors such as age, cell count, gender, smoking behavior, allergies, and specific antibody-binding peptides. This study demonstrates that both genetic and environmental exposures influence the composition of human antibody epitopes (Andreu-Sánchez et al., 2023).

In the recent past, the ToxScan library was created, which is a programmable phage display library. This was accomplished by collecting 14,430 protein sequences from the UniProt database focusing on the keywords “toxin” and “virulence factor”. This library allows an unbiased study of the connection between health or disease status and immune responses to environmental protein toxins and virulence factors (Angkeow et al., 2022). It offers an opportunity for environmental microbiome-immunome research, allowing for high-throughput and comprehensive analysis of microbial communities and antibody profiles.

#### 4.2.4 Vaccine development

Several PhIP-Seq libraries have been developed for vaccine development. The Falciparome library, which focuses on the *Plasmodium falciparum* parasite, includes all known protein sequences from two reference strains (3D7 and IT). It also contains variant sequences from key antigens, resulting in a total of 8,980 proteins. This library facilitates in-depth characterization of antibody profiles by identifying targets for naturally acquired antibody responses to *P. falciparum* (Raghavan et al., 2023). Another library, the Schistosoma mansoni antigen library, covers all 11,641 known proteins from the *Schistosoma mansoni* parasite. This library plays a crucial role in screening and characterizing vaccine-candidate antigens for *S. mansoni* (Woellner-Santos et al., 2024).

#### 4.2.5 For animal models

In addition, animal models play a crucial role in immunological research. It has been observed that a relatively low number of autoantibody studies in model organisms, such as mice has been conducted in the past. To address this gap, murine proteome-wide PhIP-Seq libraries have been introduced (Rasquinha et al., 2022; Rackaityte et al., 2023). These libraries offer new opportunities to investigate immune dysregulation and diseases in easily manageable model organisms.

## 5 Limitations and advancements of PhIP-Seq

Concurrent advancements in next-generation DNA sequencing and large-scale DNA synthesis technologies have facilitated the creation of highly multiplexed assays at a reduced cost. PhIP-Seq offers the capability to identify antibody specificity extensively, thereby offering novel insights into the host’s immune response toward the antigens. Similarly, a comprehensive understanding of the limitations of PhIP-Seq is imperative for advancing its development and application in a more rigorous and scholarly manner.

One of the major limitations of the PhIP-Seq method is the size of peptides displayed on the phage being limited to less than 100 amino acids (aa). Although the limitation of the length of peptide sequences ensures its compatibility with the short read-lengths of current NGS, it also results in its inability to capture antibodies that recognize discontinuous epitopes. To address the size limitation, the Stephen J Elledge group has developed PLATO which complements the PhIP-Seq method by using ribosome display to express intact ORFs (Xu et al., 2016). Additionally, because the length of peptides is limited, they need to overlap to cover the entire proteome comprehensively. This increases the amount of peptide that needs to be processed. An epitope-stitching approach has been proposed to splice multiple potential epitopes onto the same peptide. This method involves including immunogenic epitopes, which are preferred, and it offers a solution for displaying larger antigenic proteomes (Liebhoff et al., 2024). This method reduces the number of peptides required for phage display while effectively capturing a broader range of epitopes.

Moreover, since proteins displayed using phage-based methods such as PhIP-Seq are typically assembled and propagated in prokaryotes (*E. coli*), the proteins demonstrated in this method lack eukaryotic PTM. This could lead to false-negative results when screening for autoantibodies recognizing PTM proteins. To address this limitation, Larman’s team enzymatically modified phage-displayed polypeptides to citrullinate the relevant peptide blocks, constructing a PTM PhIP-Seq library (Román-Meléndez et al., 2021). This study offers a new approach to addressing the lack of PTM in phage-displayed peptides. However, despite these advancements, there is still a lack of incorporation of other types of PTMs, such as glycosylation, phosphorylation, deamidation, and other modifications, into PhIP-Seq platforms.

It is also important to note that PhIP-Seq is an enrichment-based assay, in which binders are serially enriched and amplified during the detection process. A drawback of this technique is the possible amplification of non-specific phages that may interfere with the recognition of the target antibody (Vazquez et al., 2022). To mitigate this issue, bioinformatics tools can be used. In data analysis, these tools play a crucial role by allowing for the effective differentiation between specific and non-specific signals, thereby improving the accuracy of results. Additionally, integrating PhIP-seq with other antibody detection techniques, such as ELISA, western blotting, or mass spectrometry analysis, can provide more comprehensive information on antibody recognition, thus reducing the impact of non-specific interference.

## 6 Future developments in PhIP-Seq

Although existing bioinformatics tools have made considerable contributions to the development of library design and data analysis in PhIP-Seq technology, there remains significant potential for further improvement.

In terms of library design, Pepsyn, which performs uniform peptide tiling across proteins remains the most commonly used tool. However, novel approaches that can overcome the drawbacks of Pepsyn including unnecessary resource investment in synthesizing, cloning, and sequencing peptides that are unreactive, limited capability in detecting reactivity to conformational epitopes, etc., are needed. One potential solution lies in designing library sequences that are capable of displaying a wider range of conformational epitopes and this can be achieved through advancements in machine learning techniques (Liebhoff et al., 2024). Additionally, the library design by Pepsyn is limited in its ability to capture conformational epitopes. To develop more efficient peptide libraries, the selected display of antigenic epitopes is essential. With the advancement of machine learning techniques, future studies are expected to design library sequences capable of displaying a wider range of conformational epitopes (Vazquez et al., 2022). Furthermore, the current library design often overlooks the potential impact of sequence mutations. Autoantigens can undergo mutations during disease evolution, which are crucial for antibody recognition and response. However, as the number of mutations increases, the size of libraries grows exponentially. Therefore, future research needs to focus on controlling the size of libraries while maintaining their diversity. This could include developing algorithms that balance the relationship between introducing mutations and library size. Alternatively, high-throughput screening techniques could be used to efficiently reduce library size and accurately capture disease-specific antibodies.

In addition, PhIP-Seq holds significant promise as a crucial tool for monitoring disease progression, especially in the context of viral infections. This technique enables the identification and profiling of specific types of immunoglobulins, offering insights into not only the dynamics of the immune response, but also their correlation to the stage of the disease. For example, IgM is typically associated with the early stages of an immune response, while IgG may suggest a later phase or a secondary exposure to the pathogen. In PhIP-Seq, protein A and protein G-coated magnetic beads were used to immunoprecipitate predominantly IgG-bound phage (Mohan et al., 2018). To investigate specific humoral responses in more detail, including isotypes or subclasses, corresponding monoclonal antibodies or reagents can be used to coat the beads. In a previous study, streptavidin-coupled magnetic beads conjugated with biotinylated omalizumab were employed to isolate and analyze IgE-specific responses. (Monaco et al., 2021). The precision of PhIP-Seq in identifying and quantifying these immunoglobulins allows for a more detailed understanding of host-pathogen interactions and subsequent immunological consequences.

Moreover, we would like to emphasize the importance of establishing a comprehensive PhIP-Seq library database and PhIP-Seq database. This centralized resource would not only serve as a comprehensive repository for antigen-antibody interaction data but also facilitate efficient data sharing and collaboration among researchers worldwide, thereby fostering global scientific cooperation.

In the analysis of PhIP-Seq data, numerous methods and models have been suggested for enrichment analysis. However, despite the availability of various approaches, there is a lack of comprehensive studies that systematically evaluate the effectiveness and performance of these methods in a comparative context. Therefore, it is essential to establish a unified evaluative framework that benchmarks these methodologies against a standardized set of criteria to advance the field.

PhIP-Seq’s ability to screen large-scale antigenic linear epitopes offers a cost-effective method for creating and characterizing antibody-specific libraries in a multi-dimensional manner. When combined with multi-omics data, it can provide a more comprehensive understanding of how antibody specificity interacts with disease mechanisms. PhIP-Seq also provides a unique perspective for investigating the complex relationships between antibody specificity and biological phenotypes. By utilizing innovative data analysis methods and fostering interdisciplinary collaborations, future studies can deepen our understanding of the immune system’s functioning and provide new insights into disease prevention, diagnosis, and treatment.

## 7 Conclusion

In summary, PhIP-Seq technology has revolutionized immunological research by providing invaluable insights into antibody specificity analysis and exploration of mechanisms underlying diseases. By integrating high-throughput DNA sequencing technology, phage display technology, and bioinformatics analysis, PhIP-Seq offers a multidimensional perspective on antibody-antigen interactions. This review comprehensively outlines the methodologies, applications, and challenges associated with PhIP-Seq technology. We also summarize the significant applications of PhIP-Seq libraries in various fields while highlighting their potential for future advancements in immunological research.

Despite the remarkable achievements of PhIP-Seq in antibody detection and disease-related studies, there are still technical and methodological challenges to be addressed. These challenges include the peptide size limitation of phage display, the absence of post-translational modifications in eukaryotes, and non-specific signal interference. Addressing these issues will require further technological innovations and interdisciplinary collaborations. Future research focused on developing new strategies for more efficient display of antigenic epitopes and exploring improved data analysis methods will be beneficial in enhancing the accuracy and reliability of antibody-specific recognition. Moreover, with continuous advancements in the fields of bioinformatics and synthetic biology, PhIP-Seq technology is expected to have a broader range of applications in personalized medicine, vaccine development, and disease prevention in the future. The integration of multi-omics data and utilization of machine learning algorithms will enhance our understanding of the relationship between antibodies and diseases. This will provide a solid foundation for the development of new therapies.

In conclusion, the development of PhIP-Seq technology not only advances immunology research but also offers new tools and methods for clinical diagnosis and treatment. With ongoing optimization of the technology and expansion of its applications, we have compelling evidence to suggest that PhIP-Seq will play a significantly more prominent role in future biomedical research.
